# Short Scales for the Assessment of Personality Traits: Development and Validation of the Portuguese Ten-Item Personality Inventory (TIPI)

**DOI:** 10.3389/fpsyg.2018.00461

**Published:** 2018-04-05

**Authors:** Andreia Nunes, Teresa Limpo, César F. Lima, São Luís Castro

**Affiliations:** ^1^Center for Psychology at University of Porto, Faculty of Psychology and Education Sciences, University of Porto, Porto, Portugal; ^2^Centro de Investigação e Intervenção Social, Instituto Universitário de Lisboa, Lisbon, Portugal

**Keywords:** TIPI, Big-Five, Portuguese, brief personality measures, psychometric properties

## Abstract

The importance of quickly assessing personality traits in many studies prompted the development of brief scales such as the Ten-Item Personality Inventory (TIPI), a measure of five personality traits (extraversion, agreeableness, conscientiousness, emotional stability, and openness). In the current study, we present the Portuguese version of TIPI and examine its psychometric properties, based on a sample of 333 Portuguese adults aged 18 to 65 years. The results revealed reliability coefficients similar to the original version (α = 0.39–0.72), very good 4-week test–retest reliability (*n* = 81, *r*s > 0.71), expected factorial structure, high convergent validity with the Big-Five Inventory (*r*s > 0.60), and correlations with self-esteem, affect, and aggressiveness similar to those found with standard measures of personality traits. Overall, our findings suggest that the Portuguese TIPI is a reliable and valid alternative to longer measures: it offers a promising tool for research contexts in which the available time for personality assessment is highly limited.

## Introduction

The Big-Five model is the most widely accepted model of personality (Woods and Hampson, 2005). It suggests five personality traits: Extraversion (to be sociable, active), Agreeableness (to be soft-hearted, trusting), Conscientiousness (to be organized, reliable), Emotional Stability (to be calm, relaxed), and Openness (to be curious, creative) (Costa and McCrae, 1992). In support of this model, consistent relationships have been found between personality traits and constructs like self-esteem (Jonason et al., 2011; Storme et al., 2016), positive and negative affect (Hofmans et al., 2008; Romero et al., 2012), and aggressiveness (Bartlett and Anderson, 2012). The expected correlations are summarized in Supplementary Table S1.

There are many instruments to measure personality traits: 240-item Revised NEO Personality Inventory (Costa and McCrae, 1992), 60-item NEO-Five Factor Inventory (Costa and McCrae, 1992), and 44-item Big-Five Inventory (BFI; John and Srivastava, 1999). However, practical constraints, such as lack of time, led to the development of briefer measures, particularly useful in large-scale assessments of differences between and within populations (Ziegler et al., 2014), when personality is not the main focus (Gosling et al., 2003). One of the shortest validated instruments to measure personality traits is the Ten-Item Personality Inventory (TIPI).

It was developed by Gosling et al. (2003), and it takes about 1 min to be completed. TIPI has become a highly influential tool in psychological research, as indicated by the number of citations of the original article (>4300, Google Schoolar). There has also been a great interest in adapting and validating TIPI for use across languages and cultural backgrounds, such as Spanish (Romero et al., 2012; Renau et al., 2013), French (Storme et al., 2016), and German (Muck et al., 2007) (for a full list, see^1^).

There is evidence suggesting that TIPI is an appropriate measure of the Big-Five model. The original TIPI (Gosling et al., 2003) showed low-to-moderate Cronbach’s alphas (α = 0.40–0.68), a typical finding in short scales (Ziegler et al., 2014), but it exhibited high temporal stability (*r*s = 0.62–0.77), strong correlations with longer personality trait measures, such as BFI (*r*s > 0.65), and patterns of correlations with other psychological variables similar to those obtained with longer measures. This has been replicated in validation studies across languages (Muck et al., 2007; Renau et al., 2013; Chiorri et al., 2015). Factorial analyses have also confirmed the five-factor structure underlying TIPI (Hofmans et al., 2008; Romero et al., 2012).

Considering the usefulness of brief measures of personality traits, this study aims to provide sound validity evidence on the Portuguese TIPI. This version was initially translated by our group (Lima and Castro, 2009, 2011) and tested by Brito-Costa et al. (2015b) with 170 male athletes (13–33 years). Another Portuguese version was developed by Carvalho et al. (2012) and tested with 404 Brazilian high-schoolers (14–20 years). Both studies have only examined TIPI’s Cronbach’s alphas and factorial structure. Therefore, further work is needed to test the Portuguese TIPI in the general population and using a more comprehensive approach to estimate its reliability and validity. Here, we evaluate TIPI based on a diverse sample (*N* = 333, 18–65 years), and we examine Cronbach’s alphas, test–retest coefficients, factorial structure using a calibration-validation design, convergence with BFI, and relationships with self-esteem, positive and negative affect, and aggressiveness.

## Method

### Participants and Procedure

Participants were 333 individuals (*M_age_* = 33.15 years, *SD* = 15.24; 78% women). A booklet including questionnaires (see below) was administered to undergraduates in classroom groups (*n* = 197). Students were then asked to take one booklet and to administer it to another person aged 40 to 65 years (*n* = 136). To assess test–retest reliability, 81 undergraduates completed TIPI again 4 weeks later. The study was approved by the Departmental Ethics Committee, and all subjects gave written informed consent in accordance with the Declaration of Helsinki.

### Materials

#### Development of Portuguese TIPI

TIPI includes two items measuring each of the Big-Five personality dimensions (Gosling et al., 2003). Within each dimension, one item represents a positive pole, the other a negative pole. Participants rate how each trait applies to themselves using a seven-point scale (1 = *strongly disagree*; 7 = *strongly agree*). The original English version was independently translated into Portuguese by two Portuguese native speakers fluent in English, and after discussion a single version was obtained (Lima and Castro, 2009, 2011). This version was piloted with 80 individuals (*M_age_* = 35.33 years, *SD* = 12.98; 49% women; Cronbach’s alphas: 0.71 for Extraversion, 0.07 for Agreeableness, 0.54 for Conscientiousness, 0.68 for Emotional Stability, 0.32 for Openness). Because alphas for Agreeableness and Openness were weaker than those of the original scale (0.40 and 0.45, respectively), we revised the translation of the corresponding items. The revised version was back-translated into English by an English-native speaker fluent in Portuguese, and all items achieved semantic equivalence with the original ones. This version was then administered to a new sample of 41 adults (*M_age_* = 21.03 years, *SD* = 2.55; 85% women). Obtained alphas were similar to those of the original English version (0.68 for Extraversion, 0.48 for Agreeableness, 0.55 for Conscientiousness, 0.67 for Emotional Stability, 0.61 for Openness). This was then considered the final version of the Portuguese TIPI, that we used in the main validation study (Supplementary Table S2), which did not include the pilot samples.

#### Additional Questionnaires

##### Big-Five Inventory (BFI)

We used BFI (John and Srivastava, 1999; Portuguese version: Brito-Costa et al., 2015a; α = 0.65–0.86) as a longer, standard measure of personality traits (44-item measure of the Big-Five dimensions of personality). Participants indicate the extent to which each trait applies to themselves using a five-point scale from *strongly disagree* to *strongly agree.*

##### Rosenberg Self-Esteem Scale (RSES)

Self-esteem was measured with the RSES, a 10-item unidimensional scale (Rosenberg, 1979; Portuguese version: Santos and Maia, 2003; α = 0.90). Participants indicate their level of agreement with a set of feelings they might have experienced in a four-point scale from *strongly disagree* to *strongly agree*.

##### Positive and Negative Affect Schedule (PANAS)

Positive and negative affect were assessed with PANAS, a 20-item measure composed of 10 positive and 10 negative feelings and emotions (Watson et al., 1988; Portuguese version: Galinha and Pais-Ribeiro, 2005; α = 0.81 for positive affect, 0.88 for negative). Participants report “how they feel in general” in a five-point scale from *nothing or very lightly* to *extremely*.

##### Aggression Questionnaire (AQ)

Aggressiveness was measured with the AQ, a 29-item measure with four subscales: Physical Aggression, Verbal Aggression, Anger, and Hostility (Buss and Perry, 1992; Portuguese version: Vieira and Soeiro, 2002; α = 0.70–0.80). Participants indicate how often they experienced the listed feelings and behaviors using a five-point scale from *never or almost never* to *always or almost always*.

### Results

#### Descriptive Statistics and Reliability Analyses

Internal consistency values for each sub-scale were similar to those found in previous studies and generally higher than those obtained by Carvalho et al. (2012) and Brito-Costa et al. (2015b). Cronbach’s alphas ranged from 0.39 for Agreeableness to 0.72 for Extraversion in Time 1, and from 0.31 for Conscientiousness to 0.79 for Extraversion in Time 2. We also found high temporal stability: test–retest coefficients varied from 0.71 for Agreeableness to 0.90 for Extraversion (**Table 1**).

**Table 1:** Descriptive statistics, reliabilities, and correlations with BFI for the TIPI dimensions.

	Original α^1^	Time 1 (*n* = 333)					Time 2 (*n* = 81)					Test–retest (4 weeks)^2^	Correlation with BFI	
TIPI dimensions		*M*	*SD*	α	*K*	*Sk*	*M*	*SD*	α	*K*	*Sk*		Convergent	Discriminant
Extraversion	0.68	4.52	1.56	0.72	–0.25	–0.72	4.38	1.46	0.79	–0.24	–0.65	0.90	0.78	0.11–0.48
Agreeableness	0.40	6.09	0.84	0.39	–1.33	2.27	5.92	0.87	0.61	–1.11	1.06	0.71	0.60	0.10–0.29
Conscientiousness	0.50	5.64	1.21	0.45	–0.84	0.21	5.25	1.11	0.31	–0.72	0.07	0.82	0.74	0.10–0.22
Emotional Stability	0.73	3.79	1.30	0.43	0.18	–0.29	3.69	1.27	0.37	0.14	–0.25	0.78	0.77	0.18–0.31
Openness	0.45	5.30	1.22	0.60	–0.68	0.27	5.20	1.02	0.48	–0.43	–0.71	0.83	0.69	0.08–0.31

*^1^From Gosling et al. (2003). ^2^Correlation between Time 1 and Time 2.*

#### Validity Analyses

##### Factor analysis

A calibration-validation design was used to test factor validity. We randomly split the total sample into a calibration sample submitted to Exploratory Factor Analyses (EFA; *n* = 133; 40% of the total sample) and a validation sample submitted to Confirmatory Factor Analysis (CFA; *n* = 200; 60% of the total sample). For EFA, we conducted a principal component analysis restricted to a five-factor solution with Varimax rotation. Results revealed that the five factors accounted for 76.01% of the total variance. The items were grouped according to the original structure of TIPI (with loadings ranging from 0.44 to 0.90), with the exception of item 2 of the Agreeableness dimension, which had a slightly higher loading on Openness (0.44 vs. 0.48, respectively). CFA was then conducted to validate the factorial structure. The proposed model did not fit the data adequately, χ^2^(25, *N* = 200) = 78.37, CFI = 0.84, SRMR = 0.07 RMSEA = 0.10, *P*(rmsea ≤ 0.05) = 0.001. After examining modification indices (MI) and factor correlations, we respecified the model. We correlated items 4 and 7 (MI = 27.23), respectively, from Emotional Stability and Agreeableness dimensions, whose items have previously been shown to cross-load (Romero et al., 2012; Renau et al., 2013; Storme et al., 2016). Also, for parsimony, the non-significant relationship between extraversion and agreeableness (*p* = 0.54) was removed. The lack of relationship between these two traits was consistently reported in prior studies with TIPI (Muck et al., 2007; Romero et al., 2012; Storme et al., 2016). This revised model fitted the data well, χ^2^(25, *N* = 200) = 46.55, CFI = 0.94, SRMR = 0.06, RMSEA = 0.07, *P*(rmsea ≤ 0.05) = 0.18, with factor loadings above 0.37, *p*s < 0.001 (**Table 2**).

**Table 2:** Principal component analysis with Varimax rotation restricted to five-factor solution and factor loadings of TIPI items.

	EFA					CFA
	E	O	A	C	ES	Factor loadings
E – Extraverted, enthusiastic	**0.85**	0.24	0.23	0.06	0.05	0.99
E – Reserved, quiet	**0.85**	0.09	–0.14	0.07	0.16	0.56
O – Open to new experiences, complex	0.22	**0.81**	0.17	–0.14	–0.17	0.66
O – Conventional, uncreative	0.20	**0.76**	–0.12	0.12	0.28	0.66
A – Critical, quarrelsome	–0.33	0.48	0.44	0.30	0.25	0.54
A – Sympathetic, warm	0.11	0.02	**0.89**	0.06	–0.10	0.39
C – Disorganized, careless	0.07	–0.22	–0.02	**0.87**	–0.02	0.37
C – Dependable, self-disciplined	0.05	0.33	0.16	**0.70**	0.12	0.88
ES – Anxious, easily upset	0.24	0.05	–0.13	0.05	**0.82**	0.49
ES – Calm, emotionally stable	–0.09	0.08	0.58	0.05	**0.65**	0.61

*A calibration-validation design was used to test TIPI’s factor validity. We randomly split the total sample into a calibration sample submitted to Exploratory Factor Analyses (n = 133; 40% of the total sample) and a validation sample submitted to Confirmatory Factorial Analysis (CFA) (n = 200; 60% of the total sample). E, Extraversion; O, Openness; ES, Emotional Stability; C, Conscientiousness; A, Agreeableness. Factor loadings higher than 0.60 are in bold. Factorial validity was tested using Exploratory Factor Analysis (EFA) and Confirmatory Factor Analysis (CFA); preliminary analyses showed that the data could be submitted to EFA (the Kaiser-Meyer-Olkin index was 0.60 and the Bartlett’s Test of Sphericity was significant, p < 0.001). For CFA, all factor loadings were statistically significant (p < 0.001).*

##### Convergence with BFI

Convergent correlations were high, ranging from 0.60 in Agreeableness to 0.78 in Extraversion (**Table 1**), and discriminant correlations were low (*r*s < 0.48).

##### Correlations with external criteria

Extraversion correlated positively with measures of self-esteem, positive affect, verbal aggression, and anger; and negatively with hostility and negative affect. Agreeableness correlated positively with self-esteem and positive affect, and negatively with negative affect and all dimensions of aggressiveness. Conscientiousness correlated positively with self-esteem and positive affect, and negatively with negative affect, verbal aggression, hostility, and anger. Emotional Stability correlated positively with self-esteem and positive affect, and negatively with negative affect, physical aggression, hostility, and anger. Finally, Openness correlated positively with self-esteem and positive affect, and negatively with negative affect and hostility (Supplementary Table S2).

To examine whether the observed correlations between TIPI and other measures matched those found for BFI, we computed *r_contrast-CV_* for each dimension (Westen and Rosenthal, 2003). Results showed that these correlations were similar between TIPI and BFI: *r_contrast-CV_* coefficients were 0.52 for Extraversion, 0.70 for Agreeableness, 0.57 for Conscientiousness, 0.83 for Emotional Stability, and 0.71 for Openness (*p*s < 0.001).

## Discussion

This study examined the psychometric properties of the Portuguese TIPI. Consistent with previous studies, we found low-to-moderate Cronbach’s alphas. Because this is a common finding when using short measures, information on temporal stability and convergence with longer measures is crucial to determine their reliability and validity (Gosling et al., 2003; Ziegler et al., 2014). Concurring with this, all five dimensions of the Portuguese TIPI displayed very good temporal stability and high convergence with BFI. These results agree with extant research using the original TIPI (Gosling et al., 2003) and translated versions (Muck et al., 2007; Romero et al., 2012; Renau et al., 2013; Storme et al., 2016).

Factorial analyses provided support to the original TIPI structure, as all items loaded on the expected dimensions. An exception was the item “critical, quarrelsome” – the negative pole of Agreeableness – that had a slightly higher loading in the Openness dimension in the EFA. Although the back-translated item matched the original one, in Portuguese, as in Spanish (Renau et al., 2013), “being critical” appears to be seen as a positive characteristic, that of being able to defend a viewpoint. This pattern of results for the Agreeableness dimension has also been obtained in other validation studies (Muck et al., 2007; Storme et al., 2016), including the cross-loading of its items (Renau et al., 2013; Storme et al., 2016). It is however important to note that all dimensions of the Portuguese TIPI displayed good temporal stability and high correlation with BFI, and exhibited a correlation pattern with external criteria as reported in previous studies using TIPI (Jonason et al., 2011) or BFI (Chiorri et al., 2015).

## Conclusion

This study developed and validated the Portuguese TIPI, which displayed psychometric properties comparable to those of the original and translated versions. Although some caution is needed when using it for individual assessments (Ziegler et al., 2014), the Portuguese TIPI offers a useful measure for research examining group differences in Portuguese-speaking participants.

## Ethics Statement

Written informed consent was obtained from all participants, and ethical approval was obtained from the Departmental Ethics Committee, Faculty of Psychology and Education Sciences, University of Porto (reference 3-1/2017). Participants were not paid for their participation, and the questionnaires were completed either in individual sessions or in class groups, using a paper-and-pencil format.

## Author Contributions

TL, CL, and SLC designed the study. CL and SLC translated the questionnaire independently. CL collected and coded the data of the first pilot study. AN collected and coded the data of the second pilot study and the main study. AN and TL analyzed and interpreted the data. All authors wrote and reviewed the manuscript, and approved its final version.

## Conflict of Interest Statement

The authors declare that the research was conducted in the absence of any commercial or financial relationships that could be construed as a potential conflict of interest.

## Footnotes

1 http://gosling.psy.utexas.edu/scales-weve-developed/ten-item-personality-measure-tipi/
