# Supplementary material for: Short Scales for the Assessment of Personality Traits: Development and Validation of the Portuguese Ten-Item Personality Inventory (TIPI)
Source: Front Psychol. 2018 Apr 5;9:461. doi: 10.3389/fpsyg.2018.00461 (PMC5895732; doi:10.3389/fpsyg.2018.00461)
Supplement: Supplementary file 1 [file Table_1.docx]

Supplementary Material

Short scales for the assessment of personality traits: Development and validation of the Portuguese Ten-Item Personality Inventory (TIPI)

Andreia Nunes, Teresa Limpo, César F. Lima, São Luís Castro^*^

*** Correspondence:** São Luís Castro: slcastro@fpce.up.pt

# Supplementary Tables

Table S1: Expected and observed correlations between TIPI scale scores and external criteria. Note. Based on Chiorri et al., 2015; Jonason et al., 2011; Marshall et al., 2015; Romero et al., 2012; Sharpe and Desai, 2001; Storme et al., 2016; Tremblay and Ewart, 2005; Zeigler-Hill et al., 2015, expected relationships between variables are presented on parentheses. The symbols (+) mean that the correlations found in previous studies were positive, (-) that they were negative, and (+/0) and (-/0) that they were ambiguous or below .20. Correlations equal to or above |.13| are statistically significant (p < .05). ^1^To facilitate interpretation the Neuroticism dimension of BFI was coded as Emotional Stability

|  |  | Extraversion | | |  | Agreeableness | | |  | Conscientiousness | | |  | Emotional Stability | | |  | Openness | | |
| --- | --- | --- | --- | --- | --- | --- | --- | --- | --- | --- | --- | --- | --- | --- | --- | --- | --- | --- | --- | --- |
|  |  | TIPI | BFI |  |  | TIPI | BFI |  |  | TIPI | BFI |  |  | TIPI | BFI^1^ |  |  | TIPI | BFI |  |
| Self-esteem |  | .29 | .38 | (+) |  | .30 | .33 | (+/0) |  | .36 | .36 | (+) |  | .48 | .51 | (+) |  | .41 | .29 | (+/0) |
| Positive affect |  | .47 | .58 | (+) |  | .25 | .28 | (+) |  | .20 | .22 | (+) |  | .30 | .33 | (+) |  | .50 | .52 | (+) |
| Negative affect |  | -.16 | -.19 | (-) |  | -.29 | -.32 | (-) |  | -.33 | -.33 | (-) |  | -.57 | -.66 | (-) |  | -.30 | -.18 | (-/0) |
| Physical aggression |  | .10 | .06 | (-/0) |  | -.29 | -.33 | (-) |  | -.10 | -.07 | (-/0) |  | -.20 | -.15 | (-) |  | -.09 | -.09 | 0 |
| Verbal aggression |  | .20 | .16 | (+/0) |  | -.37 | -.42 | (-) |  | -.14 | -.14 | (-/0) |  | -.08 | -.08 | (-) |  | .04 | .04 | (+/0) |
| Anger |  | .15 | .08 | (-/0) |  | -.48 | -.47 | (-) |  | -.19 | -.16 | (-) |  | -.45 | -.46 | (-) |  | -.13 | -.16 | 0 |
| Hostility |  | -.21 | -.27 | (-) |  | -.34 | -.41 | (-) |  | -.16 | -.23 | (-) |  | -.43 | -.46 | (-) |  | -.27 | -.20 | (-/0) |
