# Supplementary material for: Short Scales for the Assessment of Personality Traits: Development and Validation of the Portuguese Ten-Item Personality Inventory (TIPI)
Source: Front Psychol. 2018 Apr 5;9:461. doi: 10.3389/fpsyg.2018.00461 (PMC5895732; doi:10.3389/fpsyg.2018.00461)
Supplement: Supplementary file 2 [file Table_2.docx]

Supplementary Material

Short scales for the assessment of personality traits: Development and validation of the Portuguese Ten-Item Personality Inventory (TIPI)

Andreia Nunes, Teresa Limpo, César F. Lima, São Luís Castro^*^

*** Correspondence:** São Luís Castro: slcastro@fpce.up.pt

# Supplementary Tables

Table S2: Dimensions, Original Items of the TIPI and Corresponding Portuguese Translation. Note. ^1^ Reversed items, representing the negative pole of the respective dimension.

| Dimension | Original English Items | Portuguese items |
| --- | --- | --- |
|  | *I see myself as:* | *Vejo-me como uma pessoa:* |
| Extraversion | Extraverted, enthusiastic | Extrovertida, entusiasta |
| Agreeableness^1^ | Critical, quarrelsome | Conflituosa, que critica os outros |
| Conscientiousness | Dependable, self-disciplined | De confiança, com autodisciplina |
| Emotional Stability^1^ | Anxious, easily upset | Ansiosa, que se preocupa facilmente |
| Openness to Experience | Open to new experiences, complex | Com muitos interesses, aberta a experiências novas |
| Extraversion^1^ | Reserved, quiet | Reservada, calada |
| Agreeableness | Sympathetic, warm | Compreensiva, afetuosa |
| Conscientiousness^1^ | Disorganized, careless | Desorganizada, descuidada |
| Emotional Stability | Calm, emotionally stable | Calma, emocionalmente estável |
| Openness to Experience^1^ | Conventional, uncreative | Convencional, pouco criativa |
